# Cloning, Heterologous Expression, and Characterization of a βκ-Carrageenase From Marine Bacterium *Wenyingzhuangia funcanilytica*: A Specific Enzyme for the Hybrid Carrageenan–Furcellaran

**DOI:** 10.3389/fmicb.2021.697218

**Published:** 2021-08-04

**Authors:** Siqi Cao, Yuying Zhang, Guangning Chen, Jingjing Shen, Jin Han, Yaoguang Chang, Hang Xiao, Changhu Xue

**Affiliations:** ^1^College of Food Science and Engineering, Ocean University of China, Qingdao, China; ^2^Laboratory for Marine Drugs and Bioproducts, Pilot National Laboratory for Marine Science and Technology, Qingdao, China; ^3^Department of Food Science, University of Massachusetts, Amherst, MA, United States

**Keywords:** carrageenan, furcellaran, βκ-carrageenase, GH16_13, specificity

## Abstract

Carrageenan is a group of important food polysaccharides with high structural heterogeneity. Furcellaran is a typical hybrid carrageenan, which contains the structure consisted of alternative β-carrageenan and κ-carrageenan motifs. Although several furcellaran-hydrolyzing enzymes have been characterized, their specificity for the glycosidic linkage was still unclear. In this study, we cloned, expressed, and characterized a novel GH16_13 furcellaran-hydrolyzing enzyme Cgbk16A_Wf from the marine bacterium *Wenyingzhuangia fucanilytica* CZ1127. Cgbk16A_Wf exhibited its maximum activity at 50°C and pH 6.0 and showed high thermal stability. The oligosaccharides in enzymatic products were identified by liquid chromatography coupled with high-resolution mass spectrometry (LC-HRMS) and nuclear magnetic resonance (NMR) spectroscopy. It was confirmed that Cgbk16A_Wf specifically cleaves the β-1,4 linkages between β-carrageenan and κ-carrageenan motifs from non-reducing end to reducing end. Considering the structural heterogeneity of carrageenan and for the unambiguous indication of the specificity, we recommended to name the furcellaran-hydrolyzing activity represented by Cgbk16A as “βκ-carrageenase” instead of “furcellaranase”.

## Introduction

Carrageenan is a group of important polysaccharides extracted from the cell wall of red algae, which is widely applied in the food industry because of its desirable rheological properties (Chopin et al., 1999). The structures of carrageenan are complex. Depending on the occurrence of 4-linked-3,6-anhydro-α-D-galactopyranose and the sulfation pattern, carrageenan can be classified into several different types (Campo et al., 2009). Besides, carrageenan has strong intramolecular structural heterogeneity (van de Velde, 2008). Furcellaran, extracted from *Furcellaria lumbricalis*, is a kind of typical hybrid carrageenan which contained the structure consisting of an alternative β-carrageenan motif [comprising repeating 4-linked-α-D-3,6-anhydrogalactose (DA) and 3-linked-β-D-galactopyranose (G) residues] and κ-carrageenan motif [comprising 4-linked-α-D-3,6-anhydrogalactose (DA) and 3-linked-4-*O*-sulfated-β-D-galactopyranose (G4S) residues] (Knutsen et al., 1990; Supplementary Figure 1). As an important material for studying the structure–function relationship of carrageenan, furcellaran has received increasing research interest in the recent decade (Jamróz et al., 2020; Saluri et al., 2021).

Carrageenase is a group of glycoside hydrolases (GHs) that could specifically hydrolyze carrageenans (Chauhan and Saxena, 2016). They could serve as favorable tools in the structural and functional studies of carrageenans. Up to date, all reported carrageenases were found hydrolyzing β-1,4 glycosidic linkages in carrageenan. Moreover, according to their substrates, carrageenases could be divided into different types, including κ-carrageenase, ι-carrageenase, λ-carrageenase, β-carrageenase, and furcellaran-hydrolyzing carrageenases, which respectively, belonging to families GH16_17 (Shen et al., 2018; Zhu et al., 2018), GH82 (Li et al., 2017; Shen et al., 2017), GH150 (Ohta and Hatada, 2006; Guibet et al., 2007), GH167 (Hettle et al., 2019), and GH16_13 (Schultz-Johansen et al., 2018; Christiansen et al., 2020). The enzymatic activity for hydrolyzing furcellaran was discovered by Schultz-Johansen et al. (2018), based on the characterization of several novel enzymes, i.e., ph1656, ph1663, and ph1675, from the marine bacterium *Paraglaciecola hydrolytica* S66; the activity was named as “furcellaranase” in the report (Schultz-Johansen et al., 2018). Lately, two enzymes from *Colwellia echini* A3, Ce358 and Ce387, were also confirmed to degrade furcellaran (Christiansen et al., 2020). Moreover, the roles of those enzymes in the carrageenan-utilizing cascade of their originated organism were revealed. Nevertheless, the glycosidic linkage specificity of the furcellaran-hydrolyzing enzyme remained unclear. Due to the structural heterogeneity of furcellaran, two types of β-1,4 linkage present in its molecular chain, i.e., the β-1,4 linkage between β-carrageenan and κ-carrageenan from non-reducing end to reducing end (DA-Gβ1→4DA-G4S) and the β-1,4 linkage between κ-carrageenan and β-carrageenan (DA-G4Sβ1→ 4DA-G). The specificity of characterized furcellaranases on which β-1,4 linkage has not been reported.

A marine bacterium *Wenyingzhuangia fucanilytica* CZ1127^T^ (=CCTCC AB 2015089^T^ = KCTC 42864^T^) was previously isolated, identified, and sequenced (GenBank accession No. CP014224) by our lab. The bioinformatics analysis of the *W. fucanilytica* CZ1127^*T*^ genome showed the existence of an ORF (GenBank accession number WP_083194645.1; *cgbk16A*) coding a putative GH16_13 sequence. This study was aimed to clone, express, and characterize the GH16_13 protein from *W. fucanilytica*, hereinto named as Cgbk16A_Wf. Particularly, by employing Cgbk16A_Wf as a representative, the specificity of the GH16_13 furcellaran-hydrolyzing enzyme was investigated.

## Materials and Methods

### Materials

The furcellaran employed as substrate was purchased from Carbosynth (Berkshire, England). Its molecular weight (Mw) was analyzed by using high-performance size exclusion chromatography coupled with multi-angle laser light scattering and a refractive index detector (HPSEC-MALLS-RI) (Xu et al., 2016) and consequently determined as 621.3 kDa.

### Bioinformatics Analysis

The online tool dbCAN (Yin et al., 2012) and SignalP 4.1 (Petersen et al., 2011) were applied to predict possible domain structures in the protein sequence. The theoretical molecular weight and isoelectric point were calculated by ExPASy (Gasteiger, 2003). The sequence similarity of Cgbk16A_Wf and other GH16_13 family enzymes was evaluated by the BLASTP program (Altschul et al., 1997). Multiple-sequence alignments were performed by the ClustalX2 program (Thompson et al., 1997). The phylogenetic tree including Cgbk16A_Wf and all reported GH16_13 carrageenases was constructed based on the neighbor-joining method by MEGA6 (Tamura et al., 2013).

### Gene Cloning and Protein Expression

The genomic DNA of *W. fucanilytica* CZ1127 was extracted by TIANamp Bacteria DNA Kit (Tiangen, Beijing, China). The gene sequence was amplified by PCR, and the forward and reverse primers were 5′-GACACGAATTCGATAATATCACGTCTGATGATCACGTGGT T-3′ and 5′-GACACCTCGAGTTATTGAATTTTATTTTTTTGC CAAACCCT-3′. The purified product was subsequently digested by restriction enzymes *Eco*RI*/Xho*I and inserted into the pET 28a(+) vector (Novagen, San Diego, CA, United States) which contains a 6× His-tag at the N terminus. The recombinant plasmid was transformed into *Escherichia coli* BL21 (DE3) cells (Biomed, Beijing, China), and the cells were cultured in the Luria–Bertani (LB) medium (Hope Bio-Technology, Qingdao, China) containing 30 μg/ml kanamycin at 37°C until OD_600_ reached 0.4. Subsequently, the expression of the protein was induced with 0.1 mM isopropyl β-D-1-thiogalactopyranoside at 17°C for 12 h, and cells were harvested and disrupted by sonication in citrate-phosphate buffer (20 mM, pH 6.0).

The purification was conducted at 4°C, and all chromatographic steps were performed applying the ÄKTA^TM^ Prime Plus system (GE Healthcare, Uppsala, Sweden). The supernatant of the cell lysate was applied onto the HisTrap^TM^ HP columns (GE Healthcare, Uppsala, Sweden) and was eluted by 0–0.5 M imidazole in 20 mM Tris–HCl buffer (pH 8.0) with 0.3 M NaCl. The active fractions were desalted by utilizing the HiTrap^TM^ Desalting columns (GE Healthcare, Uppsala, Sweden) with 20 mM citrate-phosphate buffer (pH 6.0). The purity and Mw of purified protein were evaluated by SDS-PAGE with 5% stacking gel and 12% running gel. Protein bands were visualized by Coomassie brilliant blue. The Mw of the enzyme was calculated with markers (Page Ruler^TM^ prestained protein ladder, Fermentas, Waltham, MA, United States). The purified recombinant enzyme was employed in the following characterizations.

### The Activity Assay and Analysis of Hydrolytic Pattern

Unless otherwise stated, the furcellaran-hydrolyzing activity was assayed by incubating Cgbk16A_Wf with 2 mg/ml furcellaran in 20 mM citrate-phosphate buffer (pH 6.0) at 45°C for 10 min. The released reducing sugar in the incubation was quantified by utilizing the para-hydroxybenzoic acid hydrazide (pHBAH) method (Lever, 1972) (pHBAH was purchased from Sigma-Aldrich, St. Louis, MO, United States). One unit of the activity was defined as the amount of enzyme required to produce 1.0 μmol reducing sugar (measured as D-galactose) per minute. The protein concentration was determined by a BCA Protein Assay Kit (Beyotime Biotechnology, Shanghai, China) with bovine serum albumin as the standard.

The 0.1 U Cgbk16A_Wf was incubated with 10 mg furcellaran at 45°C for 30 min. Aliquots were taken out at time intervals and heated at 100°C for 5 min to inactivate the enzyme. The high-performance size exclusion chromatography coupled with refractive index detector (HPSEC-RID) (Agilent 1260, Agilent Technologies, Santa Cruz, CA, United States) and a TSKgel SuperAW4000 column (Tosoh Corporation, Kanagawa, Japan) were employed to analyze the products. The eluent was 0.2 M NaCl, and the flow rate was set as 0.5 ml/min.

### Biochemical Characterization

The effect of temperature on the activity was evaluated by incubating furcellaran and Cgbk16A_Wf at 20–60°C. The activity of Cgbk16A_Wf without any treatment was set as 100%, and the thermal stability was assayed by measuring the residual activity of the enzyme after being pretreated at 4, 25, 30, 45, and 50°C for 24 h.

The optimal pH was determined by incubating Cgbk16A_Wf in 20 mM various buffers with pH 3.0–11.0 (citrate-phosphate buffer for pH 3.0–6.5, NaH_2_PO_4_–Na_2_HPO_4_ buffer for pH 6.5–9.0, and Na_2_CO_3_–NaHCO_3_ buffer for pH 9.0–11.0). The pH stability was determined by pretreating Cgbk16A_Wf in buffers, respectively, at 4°C for 1 h, and the residual activity was measured after adjusting the pH back to 6.0.

The impacts of metal ions and reagents on activity were determined by adding them to the reaction mixture, respectively, with the final concentrations of 1 and 5 mM and measuring the activity ratio (%). Particularly, the effects of NaCl were assessed at different concentrations (0–0.5 M).

Kinetic constants including *Vmax*, *Km*, *Kcat* were determined by incubating Cgbk16A_Wf and furcellaran with concentrations from 0.05 to 1.60 mg/ml. The data were plotted according to the Michaelis-Menten equation by using GraphPad Prism (GraphPad Software, San Diego, CA, United States).

### LC-HRMS Analysis of Reaction Products

Different dosages of the enzyme (1–50 U) were, respectively, incubated with 50 ml furcellaran solution (2 mg/ml) at 45°C for 24 h and heated at 100°C for 5 min to inactivate the enzyme. Thereafter, the solution was centrifuged at 8,000 × *g* for 30 min after being placed at 4°C overnight and identified by the LC-HRMS technique. The LC-HRMS system was equipped with an ultra-performance liquid chromatography unit (Dionex Ultimate 3000, Thermo Fisher Scientific, San Jose, CA, United States) and the Thermo Fisher Scientific Q Exactive Orbitrap mass spectrometer (Thermo Fisher Scientific, San Jose, CA, United States) with an ACQUITY UPSEC BEH 125 SEC column (4.6 mm × 150 mm, Waters, Milford, MA, United States) for the online separation. The eluent was 20% (v/v) methanol containing 10 mM ammonium acetate, and the flow rate was set as 0.2 ml/min. The inject volume was 10 μl. The parameters for the mass spectrometer were as follows: negative ionization mode; capillary temperature, 300°C; spray voltage, 2,000 V; sheath gas pressure, 40 psi; S-lens RF level, 50 V; m/z range, 200–2,000. For assigning the detected ions, the theoretical molecular weights of carrageenan oligosaccharides were calculated with the GlycReSoft software (version 1.0) (listed in Supplementary Table 1). Besides, the “mass tolerance” was set as 5 ppm and the decimal of mass precision was set as 4.

The structural information of interested oligosaccharides was further analyzed by using LC-MS/MS. The enzymatic products were reduced by deuteration according to the previous report (Zhang et al., 2006). For MS/MS product-ion scanning, the collision energy was adjusted at 30 eV, and the other conditions were the same as above.

### NMR Analysis of Reaction Products

The final product of Cgbk16A_Wf was prepared by conducting an exhaustive enzymatic hydrolysis (incubating 100 mg furcellaran with 50 U Cgbk16A_Wf for 24 h). The product was separated on a HiLoad 26/600 Superdex 30 pg column (GE Healthcare, Uppsala, Sweden) by using the ÄKTA^TM^ Prime Plus system (GE Healthcare, Uppsala, Sweden). The eluent was 5 mM ammonium formate, and the flow rate was set as 2.6 ml/min. The purified oligosaccharides were co-evaporated with D_2_O twice by lyophilization and consequently dissolved in 500 μl D_2_O. The external standard was calibrated according to the eternal 4,4-dimethyl-4-silapentane-1-sulfonic acid (DSS) (0.00 ppm). The spectra of one-dimensional ^1^H NMR and two-dimensional correlation spectroscopy (COSY) and total correlation spectroscopy (TOCSY) were recorded by Bruker AVANCE III 600 (Bruker, Berlin, Germany) at 600 MHz under 25°C with sufficient acquisition time.

### Homology Modeling

The tertiary structure of Cgbk16A_Wf was predicted by the homology modeling server SWISS-MODEL (Guex and Peitsch, 1997). The crystal structure of κ-carrageenase PcCgkA from *Pseudoalteromonas carrageenovora* ATCC 43555 (PDB: 5OCQ) (Matard-Mann et al., 2017) was utilized as a model. The predicted structure was superimposed onto the structure of PcCgkA employing the Match Maker function of UCSF Chimera (version 1.13.1) (Pettersen et al., 2004).

### Statistical Analysis

All experiments were performed at least three times. All the data were expressed as average ± standard deviation. SPSS Statistics 19.0 (SPSS Inc., Chicago, IL, United States) was utilized to perform the Tukey’s *post hoc* test (ANOVA). The *p-*value under 0.05 was considered statistically significant.

## Results and Discussion

### Bioinformatics Analysis

**FIGURE 1:**
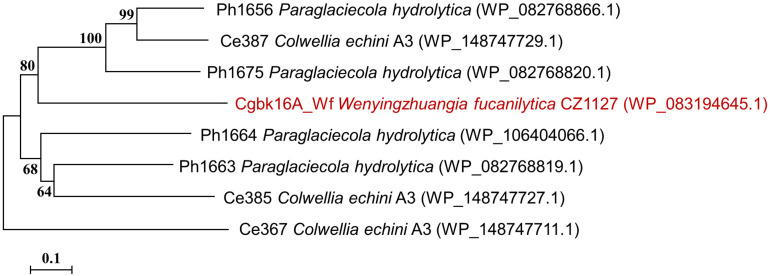
Phylogenetic tree of Cgbk16A_Wf (highlighted in red) and previously characterized GH16_13 family enzymes. The protein name, organism name, and GenBank accession number (in bracket) are successively listed in labels.

Cgbk16A_Wf was composed of 321 amino acid residues, and it contained a GH16_13 family domain (residues 60–315) without a signal peptide. The phylogenetic tree constructed by characterized furcellaranases from the GH16_13 family (Figure 1) manifested that Cgbk16A_Wf is deeply located in the branch of the GH16_13 family. The multiple-sequence alignment showed that Cgbk16A_Wf is highly conserved at ExDxxE (E179, D181, and E184 correspondingly, Supplementary Figure 2) which is the commonality of the GH16 family (Cui et al., 2017). Moreover, Cgbk16A_Wf shared the highest sequence similarity with ph1663 from *P. hydrolytica* S66 (Schultz-Johansen et al., 2018) of 40% among all reported enzymes. The above bioinformatics analysis suggested that Cgbk16A_Wf was a putative GH16_13 carrageenase.

### Cloning and Expression

**FIGURE 2:**
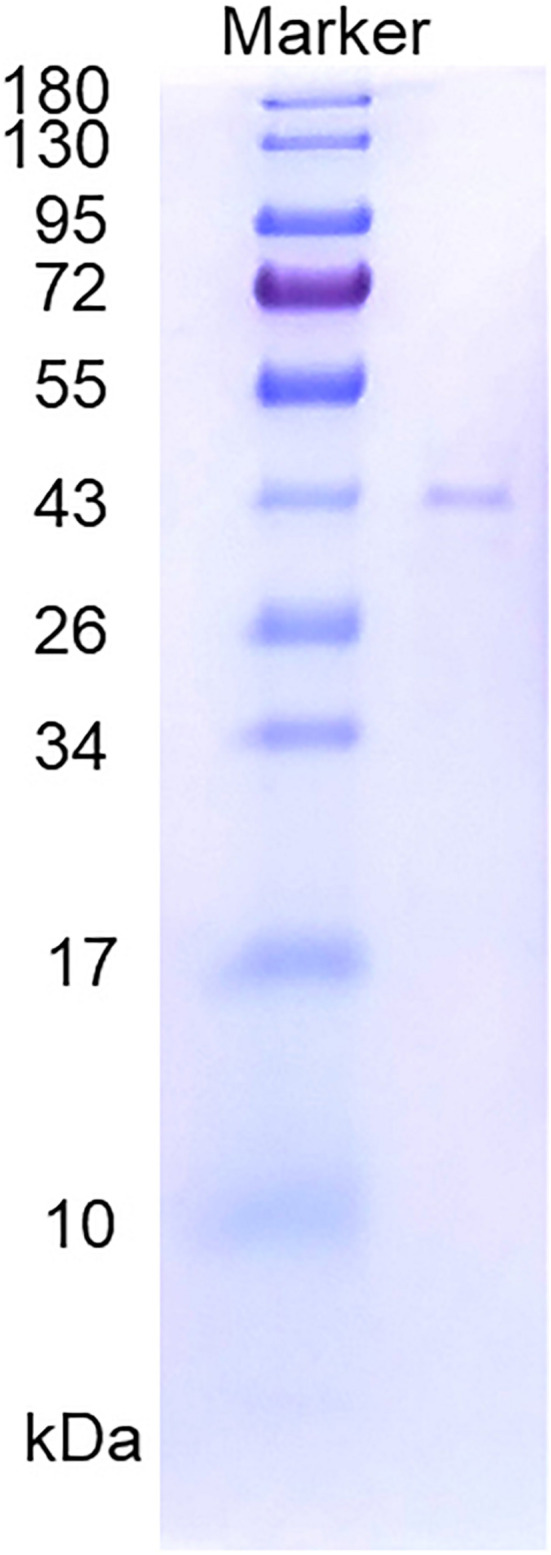
SDS-PAGE of the purified recombinant Cgbk16A_Wf.

The supernatant of the cell lysate was purified by nickel affinity chromatography, and the active fraction was eluted at an approximate imidazole concentration of 0.25 mol/l. The purified enzyme showed a single band on SDS-PAGE (Figure 2). The specific activity of the purified enzyme on furcellaran was determined as 53.75 U/mg.

**FIGURE 3:**
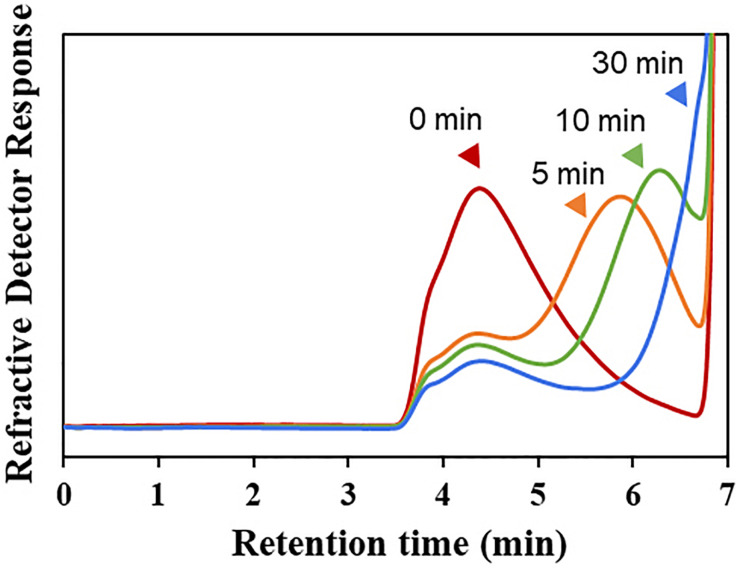
HPSEC-RID chromatograms of degradation products of Cgbk16A_Wf were examined by using the TSKgel SuperAW4000 column.

The hydrolytic pattern of Cgbk16A_Wf was analyzed by HPSEC-RID, and a significant delay of elution peak of the substrate could be observed in 5 min (Figure 3), which indicated that furcellaran was degraded rapidly by the enzyme. It suggested that Cgbk16A_Wf was an endo-acting carrageenase.

### Biochemical Characteristics of Cgbk16A_Wf

**FIGURE 4:**
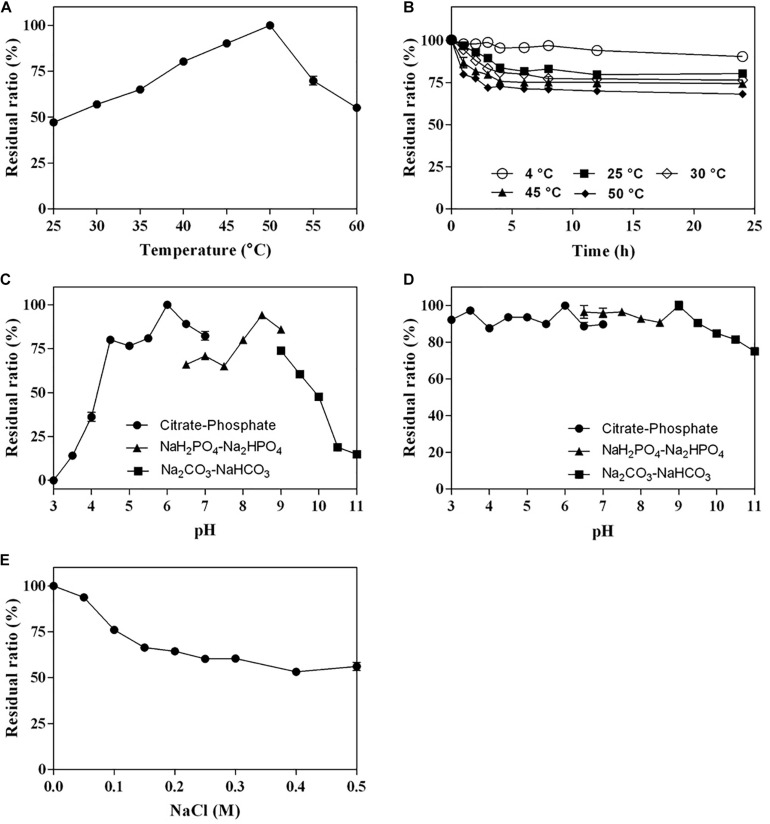
Biochemical characteristics of Cgbk16A_Wf: **(A)** effect of temperatures on enzyme activity; **(B)** thermal stability; **(C)** effect of pH on enzyme activity; **(D)** pH stability; and **(E)** impact of NaCl concentration.

The optimum temperature of Cgbk16A_Wf was 50°C (Figure 4A). The enzyme presented favorable thermal stability, and 68% of the activity could be maintained after being incubated at 50°C for 24 h (Figure 4B). Cgbk16A_Wf exhibited its maximum activity in pH 6.0 citrate–phosphate buffer (Figure 4C), and it showed more than 80% of the maximum enzymatic activity at a wide pH range from 3.0 to 10.0 (Figure 4D). Cgbk16A_Wf exhibited a high activity without NaCl (Figure 4E), suggesting that the action of Cgbk16A_Wf did not depend on NaCl, which was beneficial to the preparation of oligosaccharides. The influences of metal ions and chemicals on the activity are shown in Table 1. Cgbk16A_Wf lost most of its activity in the presence of Hg^2+^ or β-mercaptoethanol, which implied that those chemicals are able to alter the enzyme conformation and thiol-containing amino acid residues might be critical for the function of the enzyme (Sharon et al., 1998; Shen et al., 2018). The kinetics parameters *K*_*m*_, *V*_*max*_, and *K*_*cat*_ of Cgbk16A_Wf were determined as 4.81 mg/ml, 370.37 U/mg, and 270.75 s^–1^, respectively.

**TABLE 1:** Effect of metal ions and reagents (1 and 5 mmol/l) on the activity of Cgbk16A_Wf.

Compound	Relative activity (%)		Compound	Relative activity (%)	
			
	1 mM	5 mM		1 mM	5 mM
CaCl_2_	90.10 ± 2.47	95.23 ± 3.38	CuSO_4_	46.18 ± 4.45	110.45 ± 3.19
MgSO4	80.93 ± 3.35	97.98 ± 1.46	MnSO_4_	70.84 ± 4.06	64.88 ± 4.74
SDS	99.36 ± 4.77	99.54 ± 0.57	CrCl_3_	115.13 ± 4.42	102.29 ± 0.57
EDTA	122.10 ± 3.97	89.27 ± 2.21	β-mercaptoethanol	86.70 ± 3.17	71.94 ± 1.93
HgCl_2_	7.02 ± 3.91	−0.31 ± 1.15	KCl	100.73 ± 2.44	96.06 ± 1.65

### The Glycosidic Linkage Specificity of Cgbk16A_Wf

**FIGURE 5:**
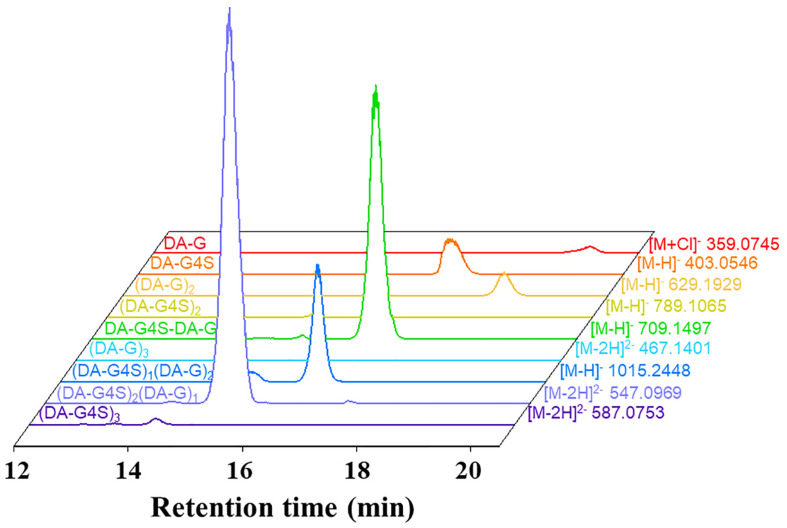
Extracted ion chromatograms of oligosaccharides in the final product.

**FIGURE 6:**
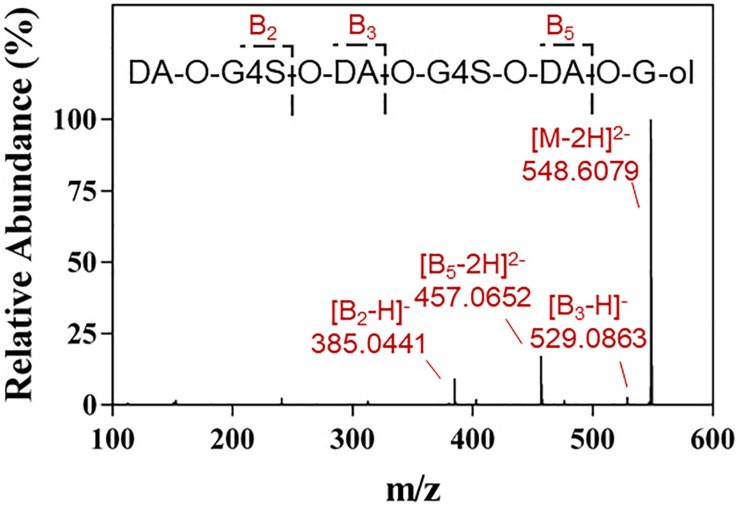
LC-HRMS/MS analysis of the hexasaccharide component (DA-G4S)_2_(DA-G)_1_ in the final product.

The final product prepared by incubating 50 U Cgbk16A_Wf with 100 mg furcellaran for 24 h was identified by LC-HRMS (Figure 5). Without considering the isomers, two disaccharides (DA-G4S, DA-G), three tetrasaccharides [(DA-G4S)_2_, (DA-G)_2_, (DA-G4S)_1_(DA-G)_1_], and four hexasaccharides [(DA-G4S)_3_, (DA-G)_3_, (DA-G4S)_2_(DA-G)_1_, (DA-G4S)_1_(DA-G)_2_] were observed. Among them, the hexasaccharide (DA-G4S)_2_(DA-G)_1_ showed the highest response which accounted for 54.4% of total oligosaccharides according to peak areas of their extracted ion chromatograms. (DA-G4S)_2_(DA-G)_1_ was thus selected and analyzed by LC-HRMS/MS. The MS/MS fragment B5 with high intensity (Figure 6) demonstrated that the G residue was situated at the reducing end, and it further indicated that the sequence of (DA-G4S)_2_(DA-G)_1_ was DA-G4S-DA-G4S-DA-G.

**FIGURE 7:**
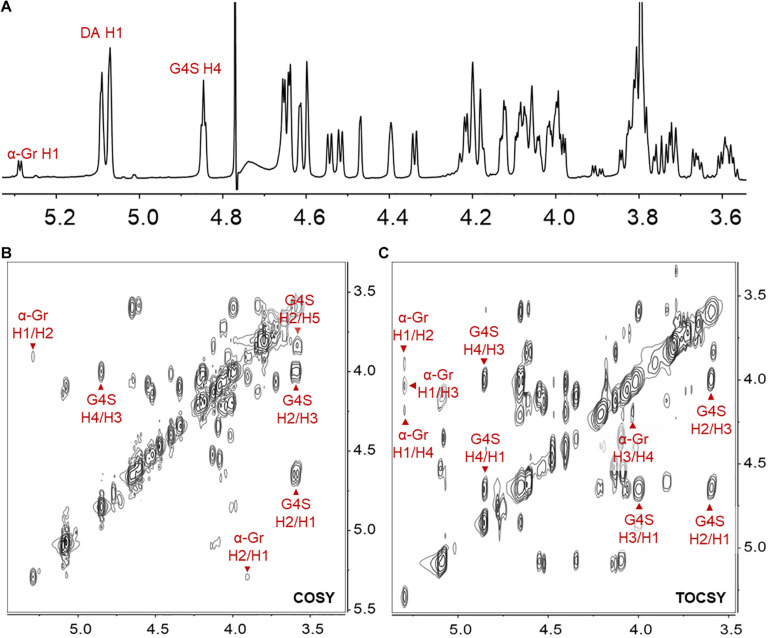
The ^1^H NMR **(A)**, COSY **(B)**, and TOCSY **(C)** spectra of the hexasaccharide (DA-G4S)_2_(DA-G)_1_ purified from the final product. The axes are ^1^H chemical shifts (ppm). The α-Gr indicated the α-G residue located at the reducing end; H1/H2 indicated the cross-peak between H-1 and H-2, etc. The ^1^H NMR, COSY, and TOCSY spectra of another hexasaccharide component (DA-G4S)_1_(DA-G)_2_ are found in Supplementary Figure 3.

**FIGURE 8:**
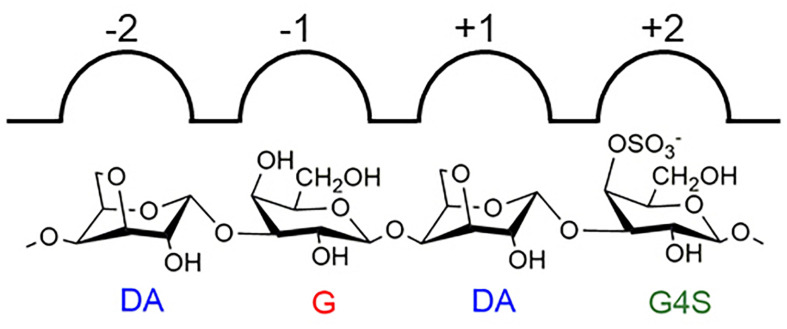
An illustration of the specificity of Cgbk16A_Wf.

Furthermore, the hexasaccharide (DA-G4S)_2_(DA-G)_1_ was prepared, purified, and identified by NMR. Several spin systems were found in its ^1^H-NMR, COSY, and TOCSY spectra. Referring to the reported data of carrageenan (Knutsen and Grasdalen, 1992; Kolender and Matulewicz, 2004), the chemical shifts of H1 to H4 of α-Gr (α-G residue located at the reducing end) were confirmed and assigned as δ 5.29, δ 3.90, δ 4.03, and δ 4.18 ppm (Figure 7). The result was consistent with that of the above LC-MS/MS analysis. Besides, the hexasaccharide (DA-G4S)_1_(DA-G)_2_ was also purified and identified by NMR (Supplementary Figure 3). The spectra confirmed that the reducing end of (DA-G4S)_1_(DA-G)_2_ was also a G residue. The corresponding assignments of chemical shift (ppm) are listed in Supplementary Table 2. It was concluded that Cgbk16A_Wf displayed a strict requirement for the G residue at its −1 subsite, i.e., the Cgbk16A_Wf specifically recognized and cleaved the β-1,4 linkages between β-carrageenan motifs and κ-carrageenan motifs from non-reducing end to reducing end (DA-Gβ1→4DA-G4S) (Figure 8).

**FIGURE 9:**
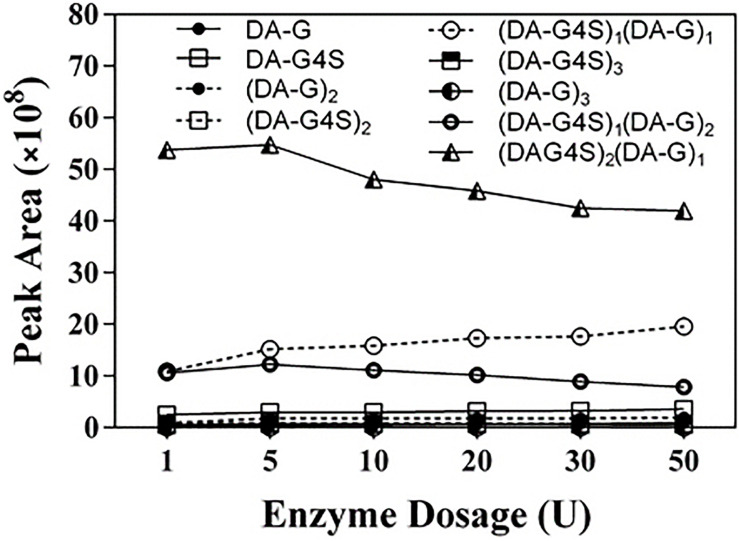
LC-HRMS analysis of products prepared by incubating 100 mg substrate with different enzyme dosages (1–50 U) of Cgbk16A_Wf for 24 h. Respective plots for disaccharides, tetrasaccharides, and hexasaccharide are found in Supplementary Figure 4.

To further investigate the action of Cgbk16A_Wf, products prepared by different dosages of the enzyme were identified by using LC-HRMS (Figure 9 and Supplementary Figure 4). Various oligosaccharides observed in low enzyme dosage (1 U) further verified that the enzyme was an endo-acting carrageenase. The hexasaccharide (DA-G4S)_2_(DA-G)_1_ consistently showed the majority in all products (Figure 9). As the enzyme dosage increased, the disaccharides and the tetrasaccharides continuously accumulated. Meanwhile, the hexasaccharides (DA-G4S)_2_(DA-G)_1_ and (DA-G4S)_1_(DA-G)_2_ increased initially and thereafter decreased. Due to the heterogeneity of the substrate, the identical composition (m/z) could be shared by several isomers with different structures. As exampled by (DA-G4S)_2_(DA-G)_1_ (molar mass as 1096.2094), an oligosaccharide with this composition might be DA-G4S-DA-G4S-DA-G, DA-G4S-DA-G-DA-G4S, or DA-G-DA-G4S-DA-G4S. Among them, DA-G4S-DA-G-DA-G4S and DA-G-DA-G4S-DA-G4S which contained the β-1,4 linkage in DA-Gβ1→4DA-G4S would be further degraded when the enzyme dosage increased, while DA-G4S-DA-G4S-DA-G was resistant to the degradation. Therefore, the observed change of hexasaccharides could be explained. Besides, it was noticed that trace oligosaccharides of (DA-G)_*n*_ and (DA-G4S)_*n*_ were produced (Figure 9); according to the specificity of Cgbk16A_Wf, we supposed that the oligosaccharides were, respectively, hydrolyzed from the non-reducing ends and reducing ends of polysaccharide chains of furcellaran rather than from its inside.

The tertiary structure of Cgbk16A_Wf was constructed (the Qmean was −6.32) by using the well-characterized κ-carrageenase PcCgkA (PDB: 5OCQ) (Matard-Mann et al., 2017) with a ligand (DA-G4S-DA-G4S) as a model, and further aligned to it. Cgbk16A_Wf shared the highest sequence identity with PcCgkA of 23.9% among all reported carrageenases with a crystal structure. It is acknowledged that κ-carrageenase could accommodate G4S at its −1 subsite. A relatively wide pocket at the −1 subsite in PcCgkA which could comfortably accommodate the sulfate group of G4S was found (Supplementary Figure 5), while in the structure of Cgbk16A_Wf, the side chain of K276 (correspondingly G258 in PcCgkA) penetrated into this pocket and spatially conflicted with the sulfate group, which implied that K276 would prevent G4S from fitting into subsite −1. It was speculated that unsulfated G residues in β-carrageenan motifs could suit the narrow −1 subsite of Cgbk16A_Wf. Nevertheless, the lysine residue was not conserved in all GH16_13 enzymes, and the molecular mechanisms behind the specificities of carrageenases in GH16 family deserve further investigation.

To the best of our knowledge, the currently reported carrageenases all specifically hydrolyzed the β-1,4 linkages rather than α-1,3 linkages in their corresponding substrates. There is only one type of β-1,4 linkage in κ-, ι-, λ-, and β-carrageenans. Therefore, the terms “κ-carrageenase,” “ι-carrageenase,” “λ-carrageenase,” and “β-carrageenase” could well reflect the substrate specificity and the glycosidic linkage specificity of these enzymes. However, as the typical hybrid carrageenan, furcellaran contains at least two kinds of β-1,4 linkages, e.g., DA-Gβ1→4DA-G4S and DA-G4Sβ1→4DA-G. Therefore, the term “furcellaranase” could only indicate its activity on furcellaran, while the glycosidic linkage specificity could not be suggested. Based on this background, we proposed that the enzymes represented by Cgbk16A_Wf with the activity of hydrolyzing the β-1,4 linkage in DA-Gβ1→4DA-G4S could be named as “βκ-carrageenase” instead of “furcellaranase.” The potential benefits of this nomenclature included the following: (1) the glycosidic linkage specificity could be precisely indicated; (2) similar to “κ-carrageenase,” “ι-carrageenase,” “λ-carrageenase,” and “β-carrageenase,” the carrageenase nature of the “βκ-carrageenase” could be intuitively conveyed; (3) considering the high heterogeneity of carrageenan, there would be more enzymes that are specifically active on hybrid carrageenans being discovered, and the nomenclature of “βκ-carrageenase” would provide a reference for the naming of these enzymes. Intriguingly, the carrageenases that could specifically hydrolyze the β-1,4 linkage in DA-G4Sβ1→4DA-G, which might be called as “κβ-carrageenase,” have not been reported yet, and we are conducting the gene exploration to discover this enzyme.

To the best of our knowledge, there are no commercial standard hybrid carrageenan oligosaccharides. In this experiment, several κ/β-hybrid oligosaccharides with high purity were obtained by using Cgbk16A_Wf, which indicated that the enzyme could serve as a powerful tool for preparing κ/β-hybrid oligosaccharides. The functional investigation on those oligosaccharides was ongoing in our lab.

## Conclusion

In conclusion, a novel GH16_13 family furcellaran-hydrolyzing enzyme Cgbk16A_Wf was cloned, expressed, and characterized. The enzyme exhibited its highest activity at 50°C and pH 6.0. It presented favorable thermal stability and maintained 68% activity after being incubated at 50°C for 24 h. Cgbk16A_Wf was an endo-acting enzyme. It displayed a strict requirement for G residue at the −1 subsite and could specifically cleave the β-1,4 linkages between β-carrageenan and κ-carrageenan from non-reducing end to reducing end. We proposed to name the activity represented by Cgbk16A_Wf as “βκ-carrageenase” instead of “furcellaranase,” which would better indicate its substrate specificity and glycosidic linkage specificity. The novel specificity, the favorable thermal stability, and the endo-acting hydrolysis mechanism indicated that Cgbk16A_Wf could serve as a powerful tool for preparing κ/β-hybrid oligosaccharides. Furthermore, it could also be utilized as a promising biotechnological tool in the structural investigation of carrageenan to reveal its heterogeneity.

## Data Availability Statement

The datasets presented in this study can be found in online repositories. The names of the repository/repositories and accession number(s) can be found below: https://www.ncbi.nlm.nih.gov/genbank/, WP_083194645.1.

## Author Contributions

SC and YC conceptualized and designed the studies and wrote the manuscript. YZ and GC designed the methodology. SC and JH performed the experimental operations. JS conducted the research and investigation. SC performed the data analyses. YC, HX, and CX revised the manuscript. All authors read and approved the final manuscript.

## Conflict of Interest

The authors declare that the research was conducted in the absence of any commercial or financial relationships that could be construed as a potential conflict of interest.

## Publisher’s Note

All claims expressed in this article are solely those of the authors and do not necessarily represent those of their affiliated organizations, or those of the publisher, the editors and the reviewers. Any product that may be evaluated in this article, or claim that may be made by its manufacturer, is not guaranteed or endorsed by the publisher.

## Supplementary Material

Supplementary Figure 1 The major structure of furcellaran.

Supplementary Figure 2 Amino acid sequence alignment of Cgbk16A_Wf with characterized GH16_13 family enzymes. Critical catalytic residues were marked by stars.

Supplementary Figure 3 The ^1^H NMR **(A)**, COSY **(B)**, and TOCSY **(C)** spectra of the hexasaccharide (DA-G4S)_1_(DA-G)_2_. The α-Gr indicated α-G residue located at the reducing end; H1/H2 indicated the cross-peak between H-1 and H-2, etc. The assignment of ^1^H chemical shifts of α-Gr and G4S were listed in Supplementary Table 2.

Supplementary Figure 4 LC-HRMS analysis of products prepared by incubating 100 mg substrate with different enzyme dosages (1–50 U) of Cgbk16A_Wf for 24 h. **(A)** Disaccharide. **(B)** Tetrasaccharide. **(C)** Hexasaccharide.

Supplementary Figure 5 The superimposition of Cgbk16A_Wf (in cyan) onto PcCgkA (in tan), focusing on the scene of −1 subsites. The ligand and the surface of PcCgkA were shown.

Supplementary Table 1 The theoretical m/z of carrageenan oligosaccharides.

Supplementary Table 2 The ^1^H chemical shift (ppm) of α-Gr and G4S residues in hexasaccharide (DA-G4S)_2_(DA-G)_1_ and (DA-G4S)_1_(DA-G)_2_.
