# Supplementary material for: Cloning, Heterologous Expression, and Characterization of a βκ-Carrageenase From Marine Bacterium Wenyingzhuangia funcanilytica: A Specific Enzyme for the Hybrid Carrageenan–Furcellaran
Source: Front Microbiol. 2021 Aug 4;12:697218. doi: 10.3389/fmicb.2021.697218 (PMC8371452; doi:10.3389/fmicb.2021.697218)
Supplement: Supplementary Figure 1 — The major structure of furcellaran. [file Data_Sheet_1.docx]

Supplementary Material

**1 Supplementary Figures**

**1.1 Figure S1**


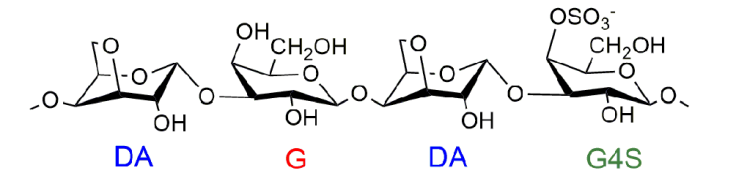


**Supplementary Figure 1.** The major structure of furcellaran.

**1.2 Figure S2**


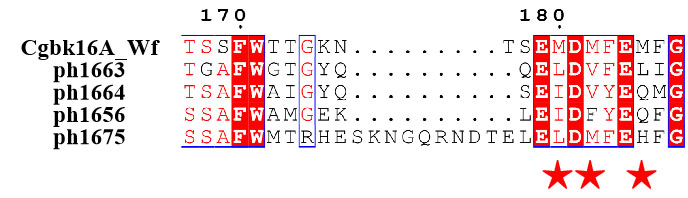


**Supplementary Figure 2.** Amino acid sequence alignment of Cgbk16A_Wf with characterized GH16_13 family enzymes. Critical catalytic residues were marked by stars.

**1.3 Figure S3**


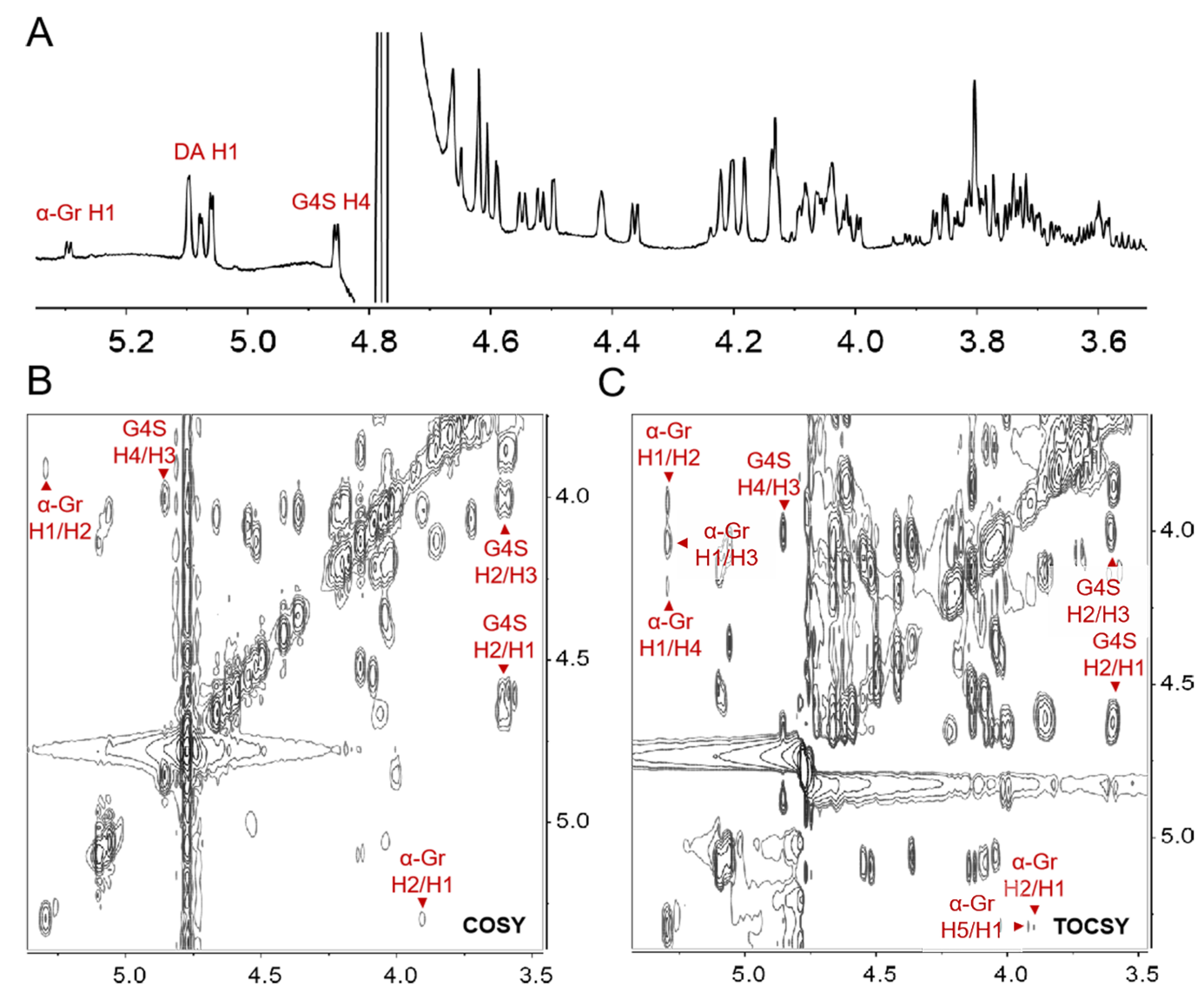


**Supplementary Figure 3.** The ^1^H NMR (A), COSY (B) and TOCSY (C) spectra of the hexasaccharide (DA-G4S)_1_(DA-G)_2_. The α-Gr indicated α-G residue located at the reducing end; H1/H2 indicated the cross-peak between H-1 and H-2, etc. The assignment of ^1^H chemical shifts of α-Gr and G4S were listed in Table S2.

**1.4 Figure S4**


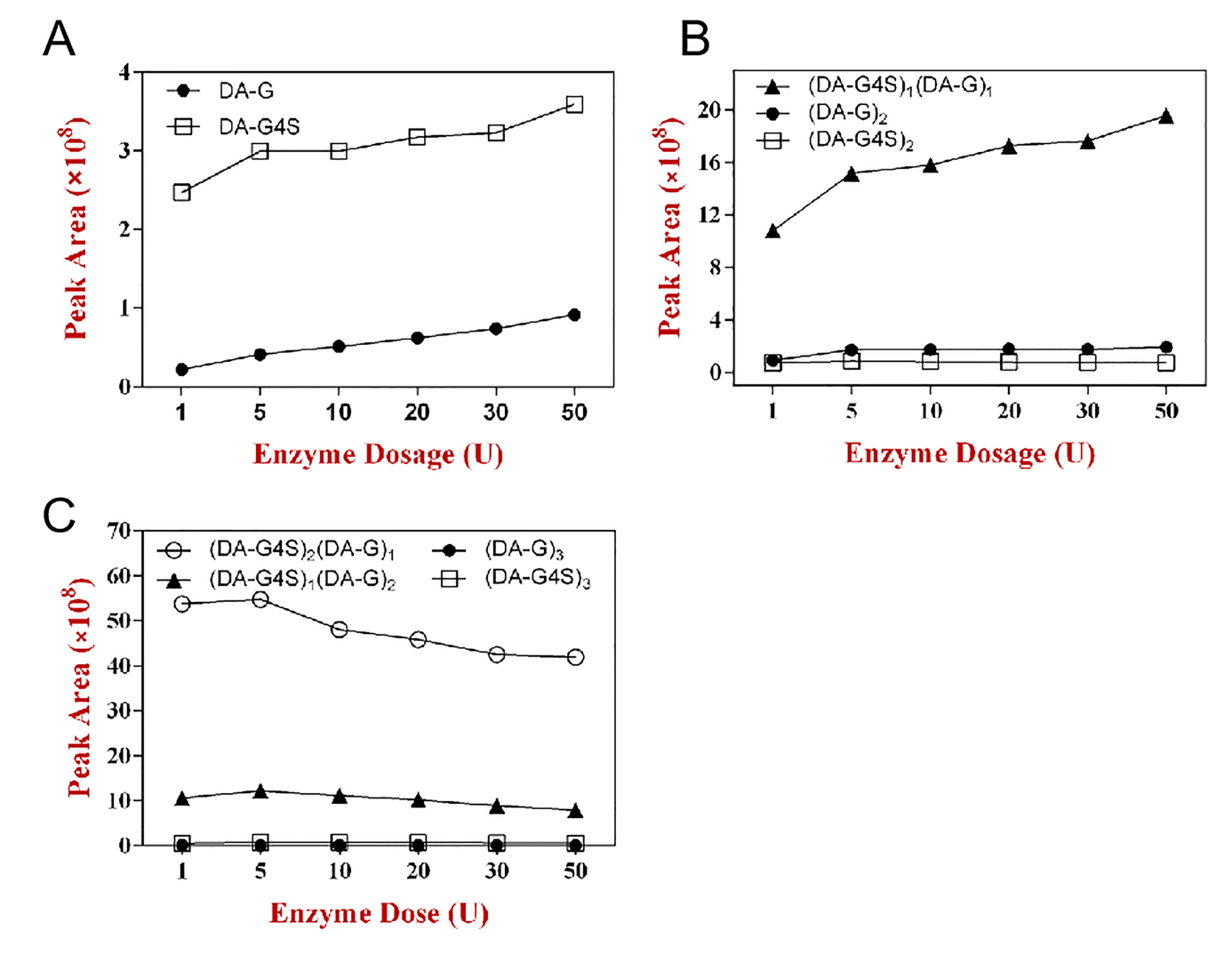


**Supplementary Figure 4.** LC-HRMS analysis of products prepared by incubating 100 mg substrate with different enzyme dosages (1-50 U) of Cgbk16A_Wf for 24 h. (A) Disaccharide. (B) Tetrasaccharide. (C) Hexasaccharide.

**1.5 Figure S5**


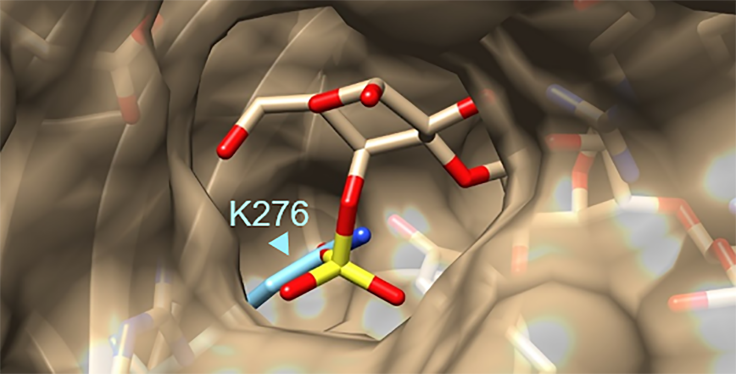


**Supplementary Figure 5.** The superimposition of Cgbk16A_Wf (in cyan) onto PcCgkA (in tan), focusing on the scene of -1 subsites. The ligand and the surface of PcCgkA were shown.

**2 Supplementary Tables**

**2.1 Table S1**

**Supplementary Table 1.** The theoretical m/z of carrageenan oligosaccharides.

| Fractions | Putative Composition | Ions | Theoretical m/z |
| --- | --- | --- | --- |
|  |  |  |  |
| dp2 | DA-G | [M+Cl]^-^ | 359.0745 |
|  | DA-G4S | [M-H]^-^ | 403.0546 |
| dp4 | (DA-G)2 | [M-H]^-^ | 629.1929 |
|  | (DA-G4S)1(DA-G)1 | [M-H]^-^ | 709.1497 |
|  | (DA-G4S)2 | [M-H]^-^ | 789.1065 |
| dp6 | (DA-G)3 | [M-2H]^2-^ | 467.1401 |
|  | (DA-G4S)1(DA-G)2 | [M-H]^-^ | 1015.2448 |
|  | (DA-G4S)2(DA-G)1 | [M-2H]^2-^ | 547.0969 |
|  | (DA-G4S)3 | [M-2H]^2-^ | 587.0753 |

**2.2 Table S2**

**Supplementary Table 2.** The ^1^H chemical shift (ppm) of α-Gr and G4S residues in hexasaccharide (DA-G4S)_2_(DA-G)_1_ and (DA-G4S)_1_(DA-G)_2_.

| Proton | | H1 | H2 | H3 | H4 | H5 | H6 |
| --- | --- | --- | --- | --- | --- | --- | --- |
| (DA-G4S)_2_(DA-G)_1_ | α-Gr | 5.291 | 3.904 | 4.031 | 4.180 | -^a^ | - |
|  | G4S nr | 4.635 | 3.585 | 4.001 | 4.846 | 3.827 | 3.609 |
| (DA-G4S)_1_(DA-G)_2_ | α-Gr | 5.295 | 3.905 | 4.037 | 4.180 | 3.919 | - |
|  | G4S nr | 4.632 | 3.605 | 4.002 | 4.855 | - | - |
| DA-G^b^ | α-Gr | 5.299 | 3.909 | 4.055 | 4.185 | 4.066 | 3.726 |
| (DA-G4S)_2_^b^ | G4S nr | 4.656 | 3.601 | 4.000 | 4.855 | 3.817 | 3.800 |

^a^ “-”: not detected.

^b^ Data are adopt from Knutsen & Grasdalen, 1992.

**Reference**

Knutsen, S. H., and Grasdalen, H. (1992). The use of neocarrabiose oligosaccharides with different length and sulphate substitution as model compounds for 1H-NMR spectroscopy. Carbohydr Res, 229, 233-244. doi: 10.1016/s0008-6215(00)90573-1
